# Electron spectroscopy for chemical analysis of liquids

**DOI:** 10.1039/d5sc09061j

**Published:** 2026-02-04

**Authors:** Lukáš Tomaník, Florian Trinter, Petr Slavíček, Bernd Winter

**Affiliations:** a Department of Molecular Physics, Fritz-Haber-Institut of the Max Planck Society Faradayweg 4-6 14195 Berlin Germany winter@fhi-berlin.mpg.de tomanikl@vscht.cz; b Department of Physical Chemistry, University of Chemistry and Technology Technická 5 16628 Prague Czech Republic

## Abstract

We present the first comprehensive, internally consistent analysis of core-level chemical shifts for aqueous-phase solutes using Electron Spectroscopy for Chemical Analysis of Liquids (ESCAL). An absolute binding-energy calibration enables high accuracy and cross-molecule comparability. The C 1s spectra of oxygenated aliphatic compounds display functional-group-specific shifts that increase with carbon oxidation state. Although these trends depart from gas- and solid-phase behavior, highlighting solvent and hydration effects, they correlate closely with calculated core-level orbital energies, providing a useful first-order predictor. We further resolve secondary, through-bond shifts over one and two bonds, the magnitudes of which depend sensitively on specific functional-group interactions (notably carboxylic acid and ketone motifs). Such element- and oxidation-state-specific structural information establishes the principles and reference data needed to build a predictive ESCAL database for liquid-phase structural and chemical analysis. The results will be contrasted with NMR studies.

## Abstract 2

Core-level chemical shifts of aqueous solutes measured by Electron Spectroscopy for Chemical Analysis of Liquids (ESCAL) show high sensitivity to molecular structure, enabling detailed resolution and making ESCAL complementary to established NMR.

## Graphical abstract

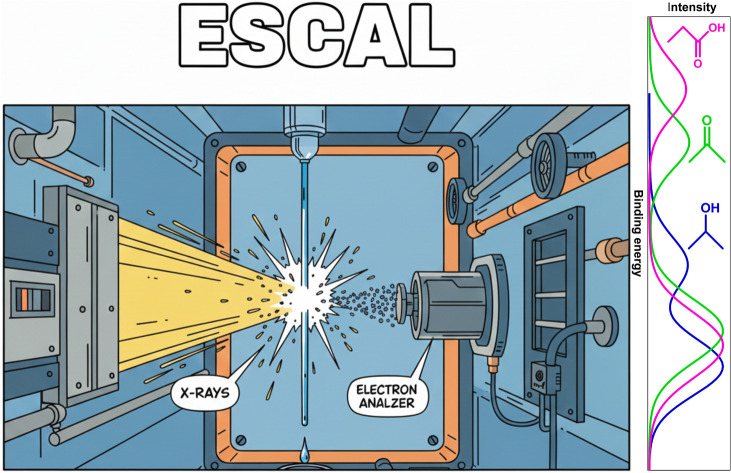

## 1 Introduction

Chemical shift is a fundamental concept in multiple spectroscopic methods, notably Nuclear Magnetic Resonance (NMR) and Electron Spectroscopy for Chemical Analysis (ESCA),^1^ also known as X-ray Photoelectron Spectroscopy (XPS). In ESCA, measured binding energies (BEs) of electrons reveal the elemental composition and, more importantly, chemical states of atoms. These BE changes, termed chemical shifts, ranging from a few meV to several eV, have proven invaluable for chemical analysis of solid-state surfaces and gas-phase molecules.^2^

The origin of ESCA chemical shifts lies in the ionization process. X-ray photons eject core-level electrons, localized on single atoms, and the recorded electron kinetic energy (KE), and hence the associated BE, reflects how strongly the electron is bound. The magnitude of the BE is governed by the net charge on the atom (initial state) and core-hole screening (final state), both of which are environment- and charge-state-dependent. Thus, any chemical modification altering charge distribution or electronic screening affects the potential field. For instance, an increased oxidation state of carbon generally increases the BE as electronegative oxygen atoms reduce electron density, increasing the positive partial charge at the carbon site, thus requiring higher photon energy for ionization. This is well-documented: gas-phase ESCA shows C 1 s BE shifts of ∼1.4 eV for alcohols and ∼3.0 eV for aldehydes/ketones compared to aliphatics,^3^ with similar trends in solid-state ESCA (∼1.6 eV and ∼2.9 eV for alcohols and aldehydes, respectively).^4^ Even secondary chemical shifts, affecting atoms one bond away, have been observed.^3,4^

While ESCA for solids and gases was developed in the 1960s, and low-vapor-pressure liquids were studied approximately one decade later,^5–7^ ESCA from volatile liquids like water became experimentally accessible only in the late 1990s due to high vacuum demands.^8^ Nowadays, this technique is known as liquid-jet photoelectron spectroscopy (LJ-PES).^9^ Additionally, combining LJ-PES with synchrotron radiation enabled precise core-level measurements and opened the door to a sharply increasing number of studies, with special emphasis on water as the most important solvent.^10^ Notably, recent advances in high-harmonic generation (HHG) laboratory sources promise broader access to such experiments.^11,12^ Despite the growing number of reported LJ-PES chemical shifts for various aqueous-phase (and non-aqueous-phase) systems, quantifying correlations between liquid-phase ESCA shifts and chemical groups or partial charges, similar to gas-phase or solid-state counterparts, has remained challenging due to the dynamic solvation environment and associated charge and energy transfers. Yet another purely experimental hurdle in LJ-PES has been accessing accurate absolute BEs associated with ill-defined potentials at the liquid-jet surface. A robust and reliable approach was introduced in 2021 by employing absolute binding-energy calibration based on low-kinetic-energy cutoffs.^13^ This development ensures BE comparability across different solutions and enables high precision in chemical shift measurements.^14^

The term ‘chemical shift’ is also central to NMR spectroscopy, which measures energy transitions of nuclear spin states in a strong magnetic field. NMR shifts arise from changes in the magnetic field caused by surrounding electrons, thus reflecting overall electron distribution.^15^ While the most commonly used proton (^1^H) NMR has a narrow chemical shift range (0–12 ppm), leading to overlapping signals in complex molecules, ^13^C NMR offers a much wider range (∼200 ppm), improving structural elucidation. The effect of electron distribution manifests in ^13^C NMR shifts of ∼10–40 ppm for aliphatic carbons, ∼55–80 ppm for alcohols, and ∼190–220 ppm for aldehydes/ketones.^16^

Despite sharing the terminology of ‘chemical shift,’ ESCA and NMR measure fundamentally different processes. It has been tempting to correlate the respective shifts, and these attempts have always shown significant variations.^17,18^ As Gelius *et al.*^19^ and Lindberg^18^ noted, while ESCA shifts can be interpreted *via* neutral-moelcule charge distributions, this is not generally true for NMR, where the dominant paramagnetic screening constant depends on excited states. Thus, a linear correlation between ESCA and NMR shifts should not be expected. Therefore, we rather view NMR and ESCA as complementary techniques for liquid-phase structural analysis.

ESCAL is enabled by experimental setups, arguably of a complexity similar to commercially available NMR spectrometers. Notably, though, the former experiments are typically performed at synchrotron radiation facilities. However, future systematic ESCAL measurements with laboratory X-ray light sources and continued improved liquid-jet delivery systems are feasible. For NMR, large libraries of documented shifts exist, helping to conveniently assign narrow peaks, measured at very high accuracy, down to hundredths of a ppm. This can be contrasted with the ∼10 meV energy resolution of ESCA from the aqueous phase, which, as we will show, is sufficient to distinguish between different molecules, charge states, and environments. We do not suggest ESCAL as a new tool for *de novo* structure determination of unknown compounds; rather, it provides element- and oxidation-state-specific constraints, complementing NMR for assignment and benchmarking. A unique feature of ESCA is its intrinsic ultrashort timescale (the time it takes to emit a photoelectron), thus providing, *e.g.*, complementary information on chemical kinetics. For example, ESCA can distinguish pseudo-equivalent nitrogen atoms in an aqueous imidazole that appear equivalent in NMR.^20^ Moreover, ESCA can directly probe elements that are not routinely accessible by conventional NMR, such as oxygen. The two methods also differ in probing depth: NMR is generally a bulk-sensitive method, while liquid-phase ESCA primarily probes the first few molecular layers. By variation of the X-ray photon energy, one can conveniently tune between surface-sensitive and more bulk-sensitive conditions. Additionally, ESCA spectra of liquids also include unique contributions of Auger-like electrons, providing specific insight into the immediate solvation environment.^21,22^

This work investigates the capability of ESCA for analysis of aqueous-phase solutes, aiming to formulate general rules for liquid-phase ESCA chemical shifts and relate them to ESCA of solids/gases. We also explore the extent of secondary shifts within molecules and address the complementarity of ESCAL to NMR. We provide the first consistent study on LJ-PES chemical shifts and their origin, deriving rules and proposing general principles.

## 2 Results and discussion

### 2.1 Chemical shifts of functional groups

**Fig. 1:**
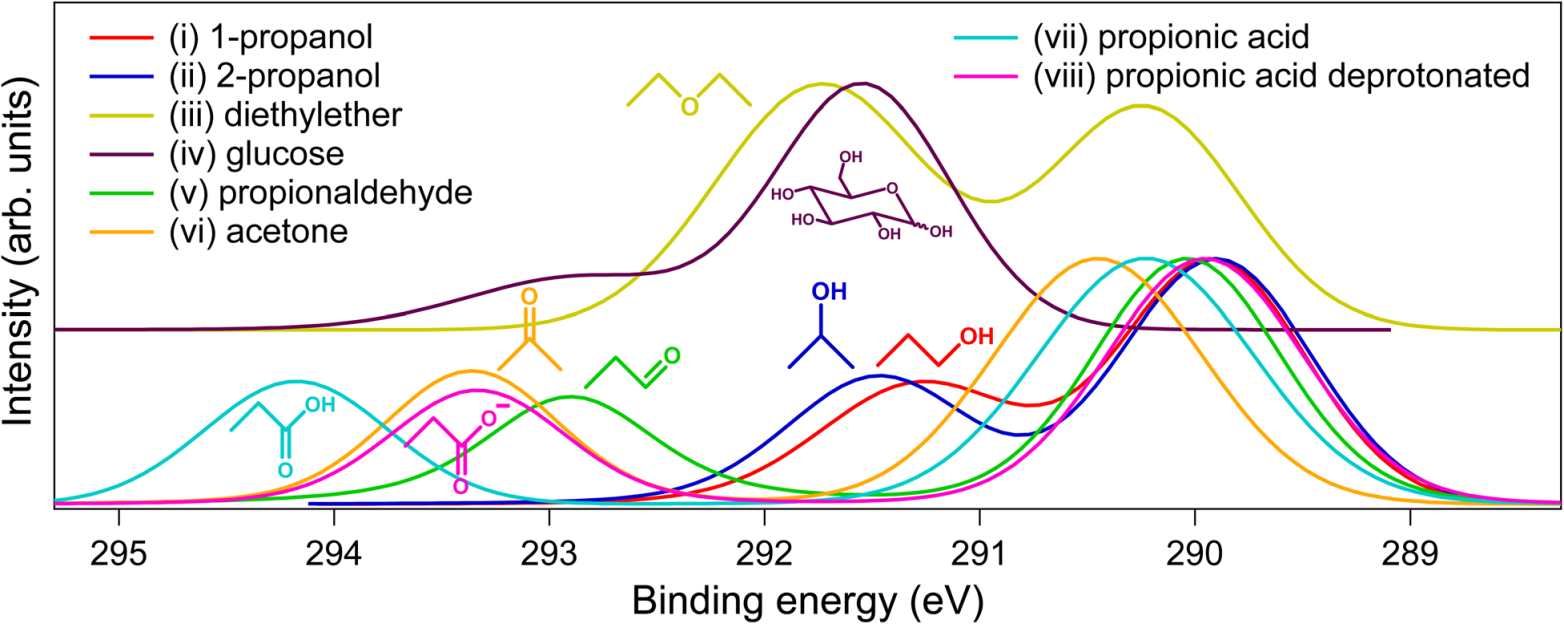
Energy-calibrated fitted C 1s photoelectron spectra of 0.5 M aqueous solutions of the compounds together with their molecular structures. Raw spectra are shown in Fig. S1 in the SI.

In this exploration of aqueous-phase ESCA, we focus on the carbon 1s electron chemical shifts in aliphatic organic compounds with oxygen-containing functional groups, a sufficient subset to demonstrate the method's capacity and lay the groundwork for future systematic extensions. The investigated functional groups include primary and secondary alcohols, ethers, hemiacetals, aldehydes, ketones, carboxylic acids, and deprotonated carboxylic acids. The molecular structures and respective photoelectron spectra are shown in Fig. 1. One observes that the peak positions differ for different compounds. The peak at ∼290 eV originates from aliphatic carbon atoms, based on experiments and calculations,^23^ while other peaks correspond to the functional groups. The widths of the peaks were similar for all the investigated compounds and, thus, not specific enough for their identification.

The BEs (peak positions) were determined as *hν* – KE and referenced to the aliphatic peak of isopropanol (–CH_3_ groups), providing self-consistent aqueous-phase ESCA chemical shifts. This reference corresponds to the lowest C 1 s BE in our set of molecules, and arises from an easily accessible compound. The results are summarized in Table 1 (columns 3 and 4). With our conservative estimate of experimental resolution (broadening from the light source and electron analyzer) of 100 meV and a much smaller peak fitting uncertainty of ∼10 meV, the shifts reliably distinguish different compounds. The largest shift is observed for carboxylic acid, significantly decreasing upon deprotonation to values similar to those of ketones. Aldehydes and hemiacetals show smaller shifts, with ethers and alcohols exhibiting even smaller shifts, followed by aliphatic groups (our zero-shift reference). Notably, ESCA distinguishes between primary and secondary alcohols (∼0.2 eV difference). These trends align with the oxidation hierarchy in organic chemistry (carboxylic acid > aldehydes/ketones > alcohols > aliphatics), where more oxidized groups bear a greater positive partial charge on a carbon atom. The deprotonated carboxylic acid shows a smaller shift due to an additional −1 charge on the molecule. The differences observed between the primary and secondary alcohols agree with our orbital energy calculations discussed below.

**Table 1:** Binding energies (BE) and chemical shifts (CS) in eV for different functional groups in aqueous solutions (aq.) measured in the given compounds in this work. Chemical shifts in gas-phase and solid-phase ESCA in eV and aqueous-phase (in deuterated water) ^13^C NMR in ppm are shown for comparison

Functional group	Compound	BE aq.	CS aq.	CS gas	CS solid	CS NMR
Aliphatics	2-Propanol	289.90	0 (arb.)	0 (arb.)	0 (arb.)	24 (ref. 24)
Alcohol primary	1-Propanol	291.28	1.38	1.4 (ref. 3)	1.55 (ref. 4)	64 (ref. 24)
Alcohol secondary	2-Propanol	291.46	1.56	1.5 (ref. 25)	1.55 (ref. 4)	65 (ref. 24)
Ether	Diethylether	291.73	1.83	1.7 (ref. 26)	1.45 (ref. 4)	66 (ref. 24)
Hemiacetal	Glucose	292.85	2.95	2.7^a^ (ref. 26)	2.93 (ref. 4)	96 (ref. 27)
Aldehyde	Propionaldehyde	292.91	3.01	3.0 (ref. 3)	2.90 (ref. 4)	203^b^ (ref. 16)
Ketone	Acetone	293.38	3.48	2.9 (ref. 3)	2.90 (ref. 4)	216 (ref. 24)
Carboxylic acid	Propionic acid	294.18	4.28	4.5 (ref. 3)	4.26 (ref. 4)	180 (ref. 28)
Deprotonated carboxylic acid	Propionic acid	293.33	3.43	—	—	185 (ref. 28)

a Acetal was used instead of hemiacetal.

b Data recorded in CDCl_3_ solvent.

Comparing LJ-PES chemical shifts to gas- and solid-phase ESCA (Table 1, columns 4, 5, and 6) reveals important distinctions. Note that the missing data for deprotonated carboxylic acid in gas/solid phases highlight the fact that this species can be readily stabilized in the aqueous phase. The correlation between gas–solid data sets is stronger than between gas–liquid or solid–liquid data, as documented by the Pearson correlation coefficient (0.992 *vs.* 0.985 *vs.* 0.985), coefficient of determination *R*^2^ (0.984 *vs.* 0.964 *vs.* 0.960), and root mean square error (0.161 *vs.* 0.242 *vs.* 0.256). In the gas phase, the overall trend of chemical shifts is similar to the aqueous phase with distinct variations (columns 4 and 5 of Table 1), with ketones being the outlier. Solid-phase data show more pronounced differences compared to the aqueous phase (columns 4 and 6 of Table 1), with particularly ketones and ethers deviating. Moreover, liquid-phase data allows distinguishing primary *vs.* secondary alcohols and aldehydes *vs.* ketones, unlike solid-state ESCA. These results underscore the unique solvent effect, indicating that liquid-phase data cannot be simply derived from gas- or solid-phase counterparts. A subsequent study using various protic and aprotic solvents of different polarities is required to understand the solvating effects resulting in ketones being an outlier in our dataset.

**Fig. 2:**
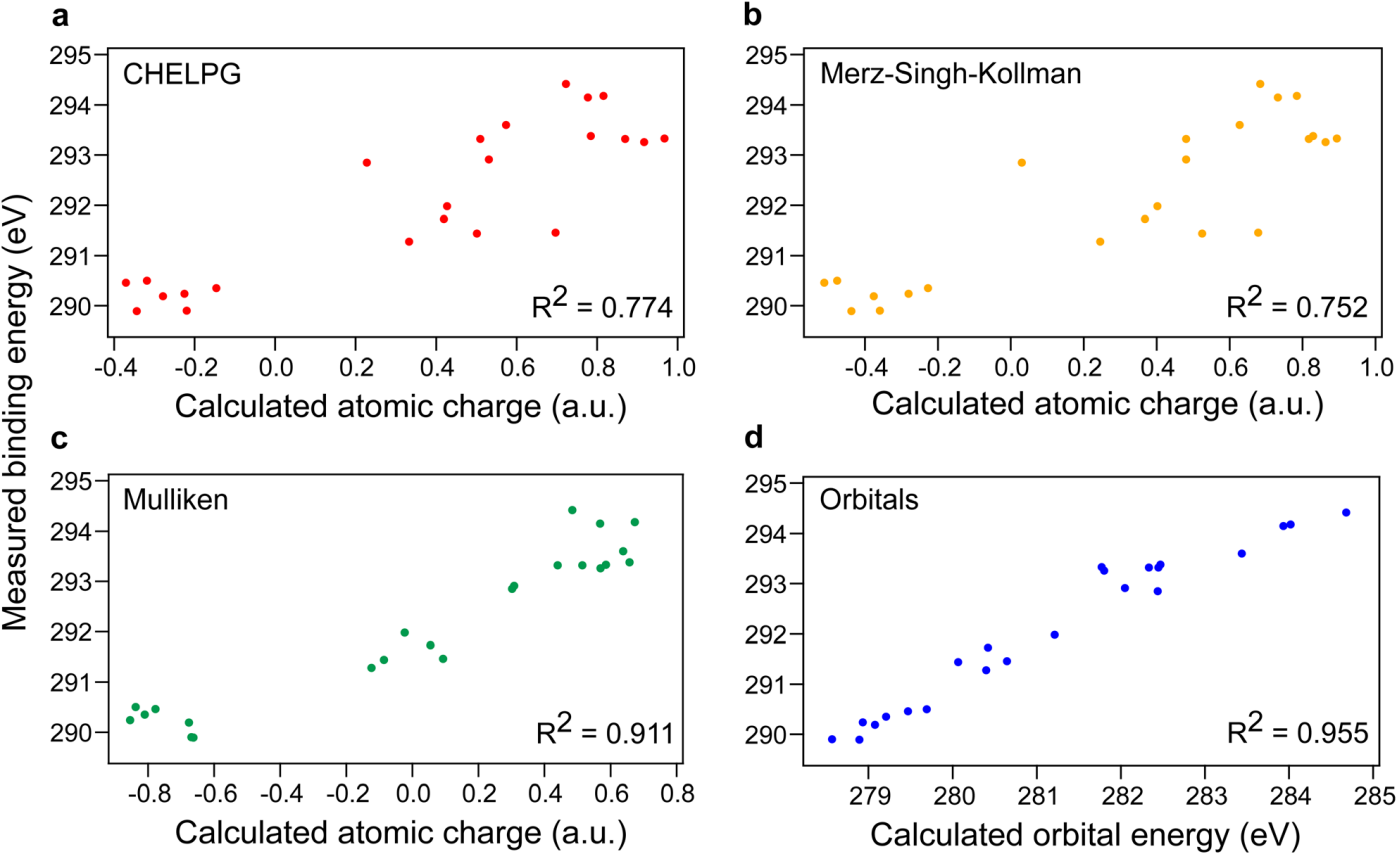
Measured carbon 1s binding energies of various carbon atoms from 0.5 M aqueous solutions of compounds shown in Fig. 1a and 4a*vs.* calculated (a): atomic charges using charges from electrostatic potentials using a grid-based method (CHELPG),^29^ (b): atomic charges using the Merz–Singh–Kollman method,^30,31^ (c): atomic charges using Mulliken population analysis,^32^ and (d): orbital energies. Methodology of calculations taken from ref. 23. *R*^2^ parameters of linear fits are shown.

In gas-phase ESCA, BE shifts are known to strongly correlate with calculated partial charges.^3^ We can ask if the same applies to the aqueous-phase data. Fig. 2 (panels a, b, and c) shows plots of our measured BEs *vs.* partial charges calculated by three different computational methods. Our results show a correlation, supporting that decreased electron density on carbon increases BEs. While calculated partial charges are method-dependent, Mulliken charges (Fig. 2c) provide better correlation than the other two tested approaches. However, these correlations are too weak for reliable BE prediction. Yet, a convincingly strong correlation was found between measured BEs and calculated C 1 s orbital energies using a published computational method^23^ (Fig. 2d), which may serve as a useful first-order estimate.

### 2.2 ESCA complementarity to NMR

**Fig. 3:**
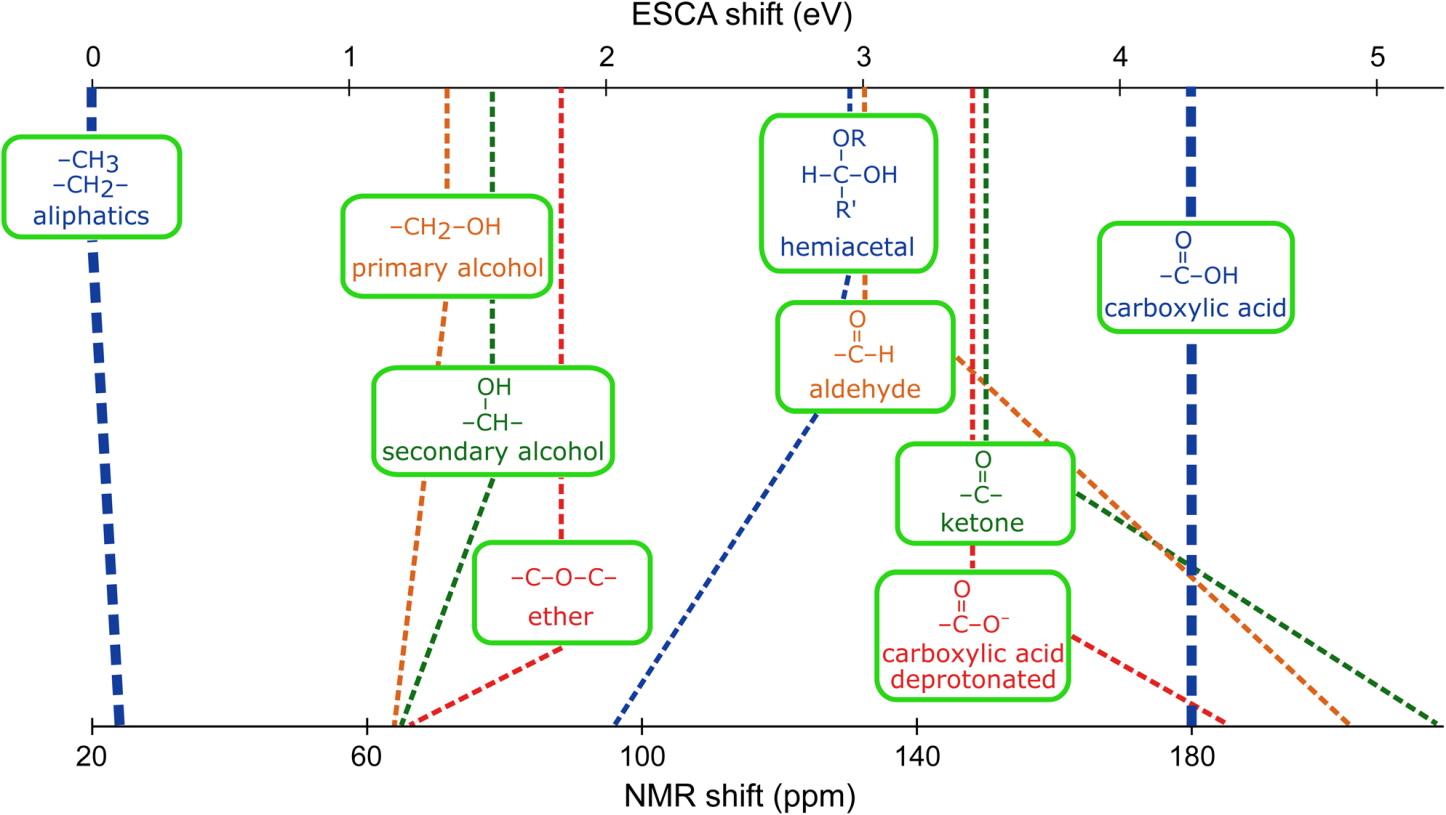
Comparison of ESCAL C 1s chemical shifts (eV) and ^13^C NMR chemical shifts (ppm) of various functional groups. The common scale is used for better visibility, with the aliphatic carbon of isopropanol and a carboxylic group as the low and high ends of the scale, respectively. Data for aqueous solutions of compounds summarized in Fig. 1 are used. Precise values of chemical shifts are summarized in Table 1.

Above, we discussed the fundamental and practical differences between ESCA and NMR. In the following, we discuss to what extent ESCAL may complement NMR studies, noting, however, that a direct 1 : 1 quantitative mapping of chemical shifts between the two methods is not generally expected. Yet, one can sometimes establish complementary directional trends, as we will show. We start by specifically focusing on chemical shifts in ^13^C NMR (Table 1, column 7) and C 1s ESCA in our set of functional groups. For better visibility, we present the data on a common axis in Fig. 3, with aliphatic carbon of isopropanol and a carboxylic acid as the low and high points, respectively. Since there is no common zero or scaling factor, we have adjusted (stretched) the NMR scale so that common trends of certain groups can be more easily identified. Overall, the comparison suggests that some groups are better distinguished in NMR while others are in ESCA. Specifically, ESCA appears to have greater resolving power for primary *vs.* secondary alcohol *vs.* ether, and for protonated (COOH) *vs.* deprotonated (COO^−^) carboxylic groups. Conversely, NMR shows a larger difference between hemiacetal *vs.* aldehyde, and ketone *vs.* deprotonated carboxylic acid. While hemiacetal and aldehyde groups exhibit very similar ESCA shifts, the respective NMR shifts are widely different and tend in different directions. Conversely, ketone and aldehyde groups show different ESCA shifts, and much higher and more similar-to-each-other NMR shifts.

### 2.3 Secondary chemical shifts

**Fig. 4:**
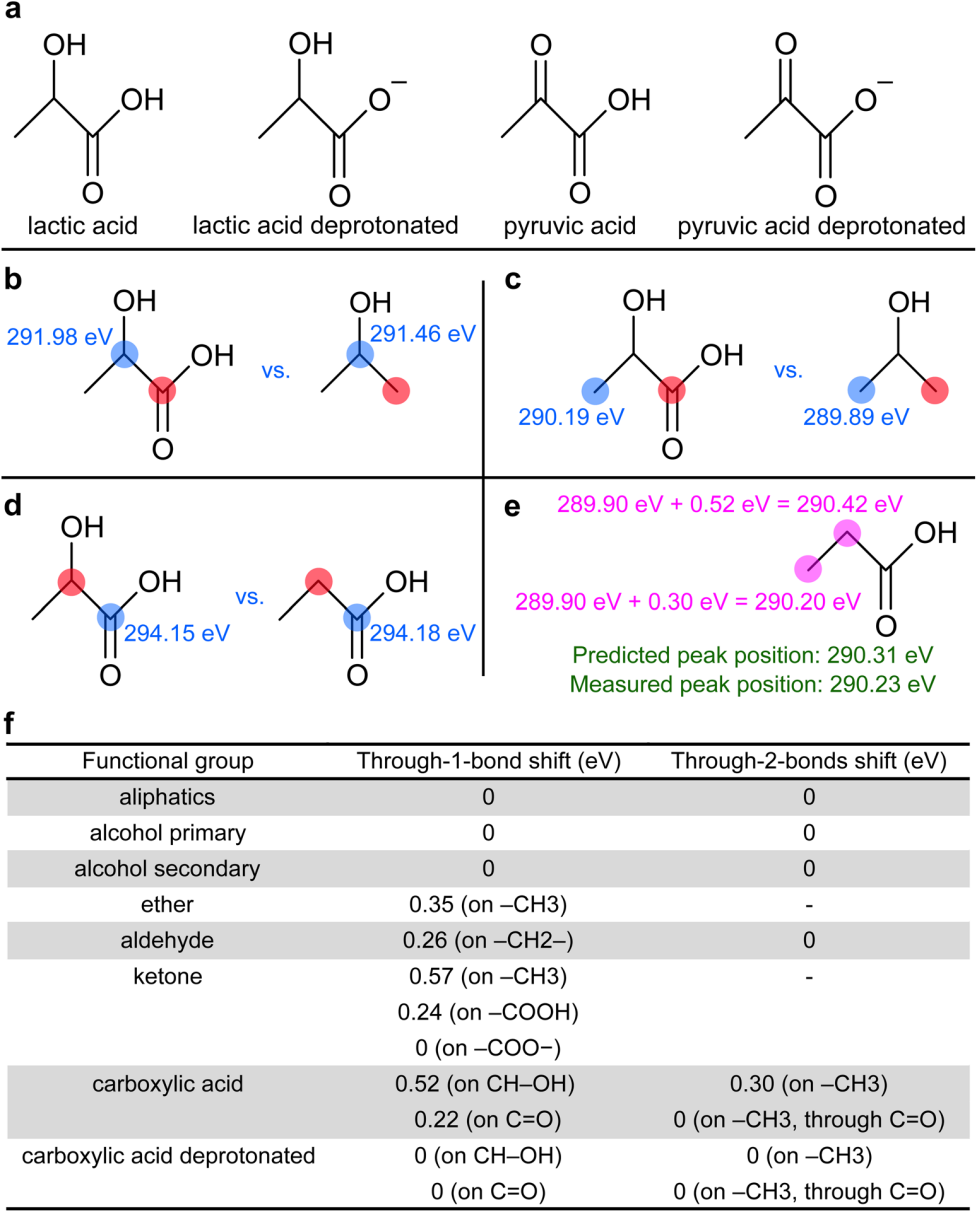
(a): List of additional molecules in our dataset containing two different functional groups to explore secondary chemical shifts. (b–d): Examples of extracting secondary C 1s chemical shifts. The monitored carbon atom is highlighted in blue, while the influence of the group highlighted in red is studied. See the main text for details. (e): Example of using extracted shifts to predict C 1 s binding energies in propionic acid. See the main text for details. (f): Extracted secondary chemical shifts in aqueous solutions caused by the presence of indicated functional groups. The affected group is given in parentheses.

We have shown the direct effect of functional groups on the ESCA shifts. However, chemical shifts can also, to a lesser extent, be introduced to adjacent atoms, one or two chemical bonds away from the functional group. These so-called secondary chemical shifts have been observed in both gas-phase^3^ and solid-state^4^ ESCA data, but they have not been explored in the liquid phase yet. This section systematically investigates this effect. We extended our molecule set by four species, each containing two different functional groups (Fig. 4a), to measure their combined effects.

Fig. 4b illustrates secondary shift extraction by comparing two compounds: one with a carboxylic group (–COOH, red), one bond away from a monitored C–OH carbon (blue), and one without (only –CH_3_). The 0.52 eV BE difference in C–OH (blue numbers) is attributed to the presence of a carboxylic acid group. Similarly, Fig. 4c shows a 0.30 eV secondary shift on a terminal –CH_3_ group (blue) caused by a carboxylic acid two bonds away (red). Conversely, Fig. 4d shows a negligible 0.03 eV difference on a carboxylic acid carbon (blue) due to an adjacent C–OH group (red), below our 0.1 eV experimental resolution, indicating no secondary shift from C–OH on carboxylic acid carbon. A similar analysis was performed for other molecules presented in Fig. 1 and 4a, with all BE values being detailed in Fig. S2 in the SI. All extracted secondary chemical shifts are summarized in Fig. 4f. Aliphatic groups and alcohols generally do not introduce secondary shifts on other carbon atoms. In contrast, highly oxidized groups like carboxylic acids and ketones cause strong secondary shifts (up to 0.57 eV), detectable even two chemical bonds away.

We tested these derived shifts on the aliphatic carbons of propionic acid (highlighted in purple, Fig. 4e), which appear as a single peak in the photoelectron spectrum (Fig. 1). Applying derived shifts from Fig. 4f, the –CH_2_– group next to the carboxylic group should shift by 0.52 eV from the unaffected aliphatic reference (289.90 eV) to 290.42 eV. The terminal –CH_3_ group should shift by 0.30 eV to 290.20 eV. Combining these yields a predicted peak position of 290.31 eV, which aligns well with the measured peak position of 290.23 eV.

Secondary chemical shifts summarized in Fig. 4f allow for several more conclusions unique to the aqueous phase. (A) ESCAL shifts are not universal nor strictly additive; they depend on the interacting groups and their mutual position. This is illustrated by different shifts caused by ketones or by carboxylic acids (Fig. 4f). (B) We can classify functional groups of similar properties: (i) aliphatic groups and alcohols do not cause secondary shifts, and their BEs are largely affected by the presence of other groups. (ii) Deprotonated carboxylic acids neither affect nor are they affected by other groups in this set. (iii) Aldehydes cause secondary shifts through one chemical bond. (iv) Ketones and carboxylic acids cause strong secondary chemical shifts, even through two bonds.

Overall, these physically meaningful chemical shifts clearly propagate through one and sometimes two chemical bonds and are specific to interacting group combinations. A larger, consistently acquired dataset could significantly extend this matrix, providing strong predictive power and serving as a useful lookup reference for any LJ-PES core-level measurements. Importantly, future additions must use the low-kinetic-energy cutoff method,^13^ as applied here, and detailed in the Methods section. For consistency, we suggest keeping isopropanol as a reference.

### 2.4 Extension to other elements

**Fig. 5:**
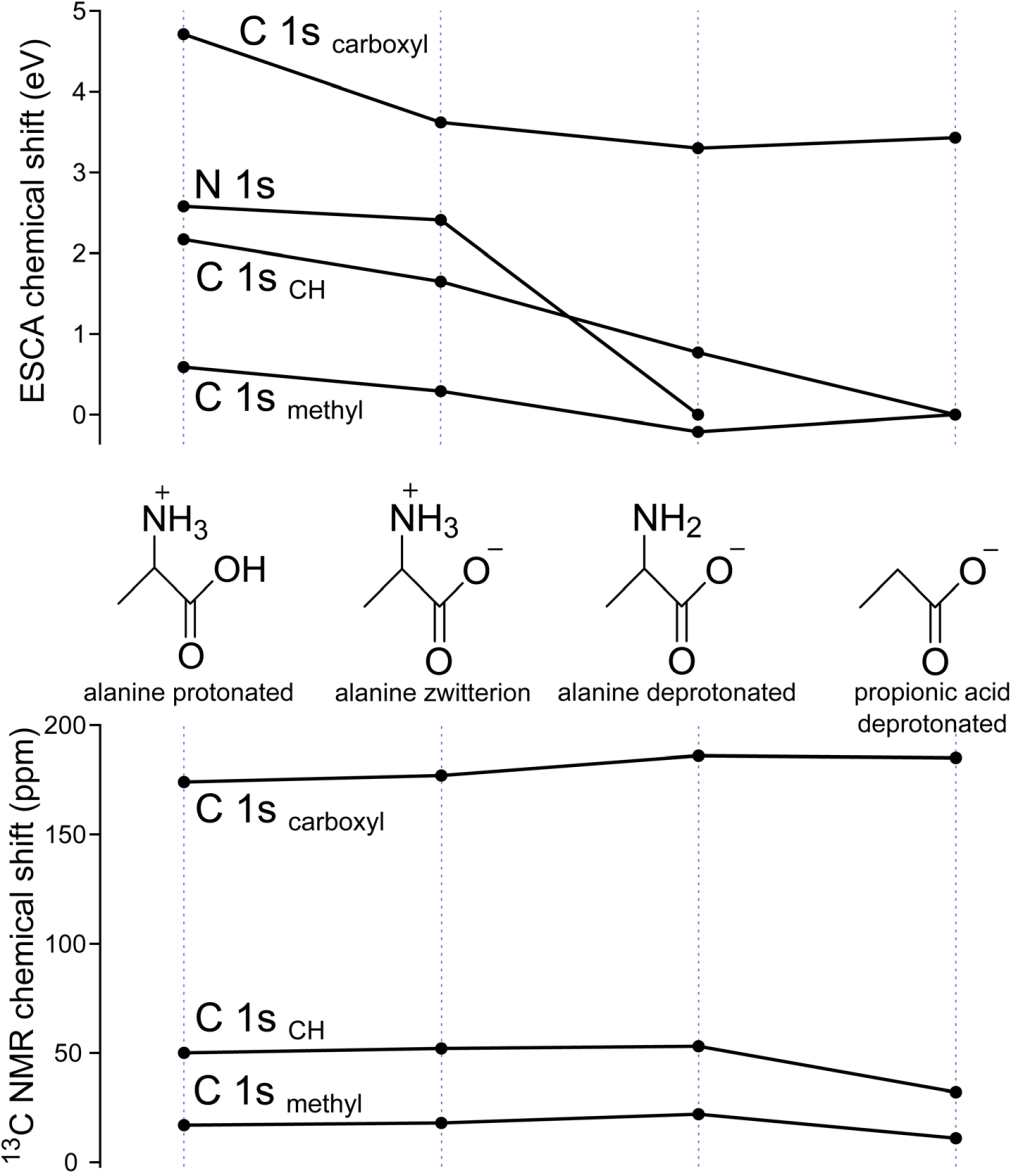
ESCAL and NMR chemical shifts of aqueous solutions of three different acid-base forms of alanine and a deprotonated form of propionic acid. Upper panel: ESCA shifts of nitrogen 1 s binding energies (referenced to the BE of –NH_2_ in deprotonated alanine) and of carbon 1 s BEs, measured in this work. Lower panel: ^13^C NMR chemical shifts taken from ref. 16.

So far, we have focused on the C 1s spectra of aliphatic compounds with oxygen-containing groups, but the principles demonstrated here can be extended to other groups and elements. As an example, we show the data for the amino acid alanine, extending our exploration to nitrogen atoms in amino groups. We measured carbon 1s as well as nitrogen 1s photoelectron spectra of alanine at three different pH values to produce three different protonation forms and compared the results to propionic acid (Fig. 5). We first focus on N 1s shifts (Fig. 5, upper panel). The protonated and deprotonated amine groups (−NH_3_^+^/–NH_2_) are easily distinguishable with the shift in BEs of ∼2.4 eV, in line with other works on LJ-PES of amino acids.^33^ Next, our focus is on C 1s shifts, shown in the upper panel of Fig. 5. The comparison with propionic acid reveals secondary chemical shifts introduced by the presence of the amine group, propagating through one and two chemical bonds. The protonated amine group (−NH_3_^+^) causes very strong shifts of C 1s to higher BEs. Notably, the presence of the –NH_2_ group increases the BE of the carbon atom through one chemical bond but decreases the BEs of carbons through two chemical bonds.

For comparison, we present the respective ^13^C NMR chemical shifts shown in the lower panel of Fig. 5. As already seen in Fig. 3, there is no common scaling factor to meaningfully connect the chemical shifts from the two methods. Nevertheless, we identify directional trends, qualitatively similar for each protonation state of alanine (and especially protonated *vs.* zwitterionic species). However, NMR resolves even the subtle change of 1.7 ppm between the protonated and deprotonated carboxylic groups that can still be reliably measured. Despite its much higher energies, ESCA has far poorer absolute energy resolution (meV–eV) than NMR (10^−15^–10^−13^ eV) because core-hole lifetime broadening fundamentally limits XPS, whereas NMR nuclear spin transitions are extremely long-lived.

## 3 Conclusions

We investigated whether chemical shifts in LJ-PES (liquid-phase ESCA or ESCAL) can be used similarly to ESCA of solids and gases. We recorded C 1s core-level photoelectron spectra of aqueous solutions of oxygen-containing aliphatic organic compounds and extracted absolute and accurate BEs of different carbon 1s electrons. Our results demonstrate detectable chemical shifts specific to functional groups. The trends in liquids differ from those in the gas or solid phase and highlight unique solvent effects. An observed strong correlation between experimental ESCAL shifts and calculated orbital energies enables a predictive first-order estimate of the former. ESCA and NMR chemical shifts are found to provide similar sensitivity in resolving molecular structure details, making ESCA a truly powerful alternative method to well-established NMR. The two techniques differ primarily in underlying processes, and hence observables, and time scales. Furthermore, a unique ESCA feature manifests in the capability to probe through-bond chemical shifts up to several chemical bonds away from the functional group in aqueous solutions. These secondary chemical shifts vary in strength based on functional-group type and are not universal, but dependent on specific group combinations. Observed trends enable the creation of a predictive database. This work provides the first consistent data subset, laying essential groundwork for determining empirical rules for predicting and interpreting LJ-PE spectra, similar to ESCA of gases or solids. With the expected increasing availability of liquid-phase ESCA setups, this approach can be extended to other functional groups and elements in future studies. Yet, despite its enormous sensitivity to structure determination, the performance of ESCAL experiments is currently less convenient than NMR analysis.

## 4 Methods

### 4.1 Experimental

Liquid-jet photoelectron spectra were measured at the P04 soft X-ray beamline of synchrotron PETRA III (DESY, Hamburg, Germany). We used circularly polarized light, a planar Pt-coated mirror, a laminar grating with 1200 lines/mm and 9 nm groove depth, and a vertical exit-slit size of 100 µm.^34^ The exact photon energy of 480.46 eV (April 2024) and 480.07 eV (December 2024) was calibrated using a precisely known liquid water valence 1*b*_1_ peak position of 50 mM NaCl aqueous solution of 11.33 eV.^13^ The beamline resolution with the described settings was 67 meV.

We used our EASI^35^ setup for the measurements. A liquid microjet under vacuum was produced using a fused-silica nozzle with an inner diameter of ∼30 µm. Solutions were delivered by a high-performance liquid chromatography (HPLC) pump at a constant flow rate of ∼1 mL min^−1^. A small metallic tube, placed in the main polyether ether ketone (PEEK) liquid-delivery line, enables electric grounding of the liquid or application of a defined bias voltage. The vacuum of ∼10^−4^ mbar in the interaction chamber was maintained using a set of scroll and turbomolecular pumps. The injected liquid was collected in liquid-nitrogen-cooled traps at the far end of the interaction chamber. The laminar-flow part of the jet, typically extending 5–10 mm from the nozzle, was irradiated by the X-ray beam in a perpendicular orientation. Photoelectrons were detected in a backward-scattering detection geometry, corresponding to an angle of 130° with respect to the light propagation direction, *i.e.*, near magic angle, thus minimizing any differential sensitivity to the photoelectron angular distributions.^35^ The detection of photoelectrons was done using a hemispherical analyzer. The analyzer slit of 0.5 mm and pass energy of 50 eV correspond to the analyzer resolution of 63 meV. Thus, the total resolution as a combination of beamline and analyzer resolution is 92 meV.

The reported binding energies (BE) in this work refer to the absolute energy scale (with respect to the vacuum) and were determined using a difference between the respective peak position (*P*) and the low-kinetic-energy cutoff (*C*), using precisely calibrated photon energy (*hν*), BE = *hν* – (*P*–*C*). We applied a bias voltage of −32 V to the liquid (accelerating the photoelectrons) in all measurements. This procedure of determining absolute BEs in liquids has been detailed in ref. 13.

The 0.5 M solutions were prepared by dissolving the respective amount of solid in or mixing the respective amount of liquid with MilliQ water (18.2 MΩ cm^−1^). We used the following commercially available chemicals: Propan-1-ol (Thermo Fisher Scientific, ≥99% purity), 2-Propanol (Sigma-Aldrich, ≥99.8% purity), Diethyl ether (Merck, for analysis), D-(+)-Glucose (Alfa Aesar, ≥99% purity), Propionaldehyde (Thermo Fisher Scientific, ≥99% purity), Acetone (Carl Roth, ≥99.8% purity), Sodium propionate (Sigma-Aldrich, ≥99% purity), d-Lactic acid (Biosynth), Pyruvic acid (Sigma-Aldrich, ≥98% purity), and l-Alanine (Thermo Fisher Scientific, ≥99% purity). The respective protonation forms of propionic acid, lactic acid, pyruvic acid, and alanine were prepared by adjusting the solutions' pH (measured with a Mettler Toledo FiveEasy pH meter^1^) by adding hydrochloric acid (32%, Merck, for analysis) or sodium hydroxide (Sigma-Aldrich, ≥95% purity). Moreover, a small amount of sodium chloride (Sigma-Aldrich, ≥99% purity) corresponding to 50 mM concentration was added to non-ionic solutions to ensure the sample's conductivity.

### 4.2 Calculations

Atomic charges and orbital energies for each compound (and each protonation state) were calculated with a single optimized structure based on our previous work.^23^ The optimizations were performed using the hybrid functional based on the B3LYP density functional with the Coulomb-attenuating method, together denoted as CAM-B3LYP,^36^ and Pople's basis set 6-31+G*. To model hydration, we used the Polarizable Continuum Model^37,38^ (PCM), with universal force field (UFF) atomic radii^39^ and an electrostatic scaling factor *α* = 1.1. All the optimized structures were confirmed to be real minima by a frequency analysis. We performed Mulliken population analysis and calculated electrostatic potentials using the Merz–Singh–Kollman scheme and the CHELPG scheme. The calculations were done using Gaussian 09, revision D.01 (ref. 40).

## Author contributions

P. S. conceived the study. L. T. designed the study. L. T., F. T., and B. W. prepared and performed the experiments. L. T. analyzed the data. L. T. performed the calculations. L. T., P. S., and B. W. wrote the paper with feedback from all authors. B. W. supervised the project. F. T. and B. W. provided experimental resources. P. S. provided computational resources.

## Conflicts of interest

There are no conflicts to declare.

## Supplementary Material

SC-OLF-D5SC09061J-s001

SC-OLF-D5SC09061J-s002

## Data Availability

Raw experimental data, figure traces, and computational input and output files have been deposited at Zenodo, https://doi.org/10.5281/zenodo.17043535. Supplementary information (SI): Raw photoelectron spectra and overview of C 1s and N 1s binding energies measured in this work. See DOI: https://doi.org/10.1039/d5sc09061j.
