# Supplementary material for: Electron spectroscopy for chemical analysis of liquids
Source: Chem Sci. 2026 Feb 4;17(12):6156–64. doi: 10.1039/d5sc09061j (PMC12869189; doi:10.1039/d5sc09061j)
Supplement: SC-017-D5SC09061J-s001 [file SC-017-D5SC09061J-s001.pdf]

Supplementary Information (SI) for Chemical Science.
This journal is © The Royal Society of Chemistry 2026

Supplementary Information: Electron Spectroscopy for Chemical Analysis of Liquids

Lukáš Tomaník,*,†,‡ Florian Trinter,† Petr Slavíček,‡ and Bernd Winter*,†

†*Department of Molecular Physics, Fritz Haber Institute of the Max Planck Society, Faradayweg 4-6, 10587 Berlin, Germany*

‡*Department of Physical Chemistry, University of Chemistry and Technology, Technická 5, 16628 Prague, Czech Republic*

E-mail: tomanikl@vscht.cz; winter@fhi-berlin.mpg.de

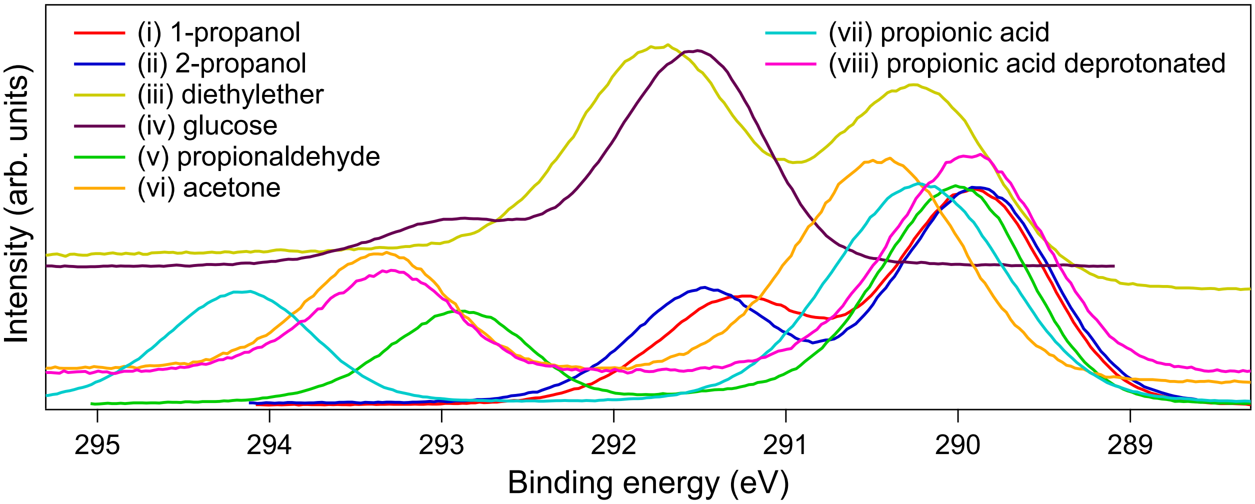

Figure S1: Raw energy-calibrated C 1s photoelectron spectra of 0.5 M aqueous solutions pertaining to Fig. 1 in the main text.

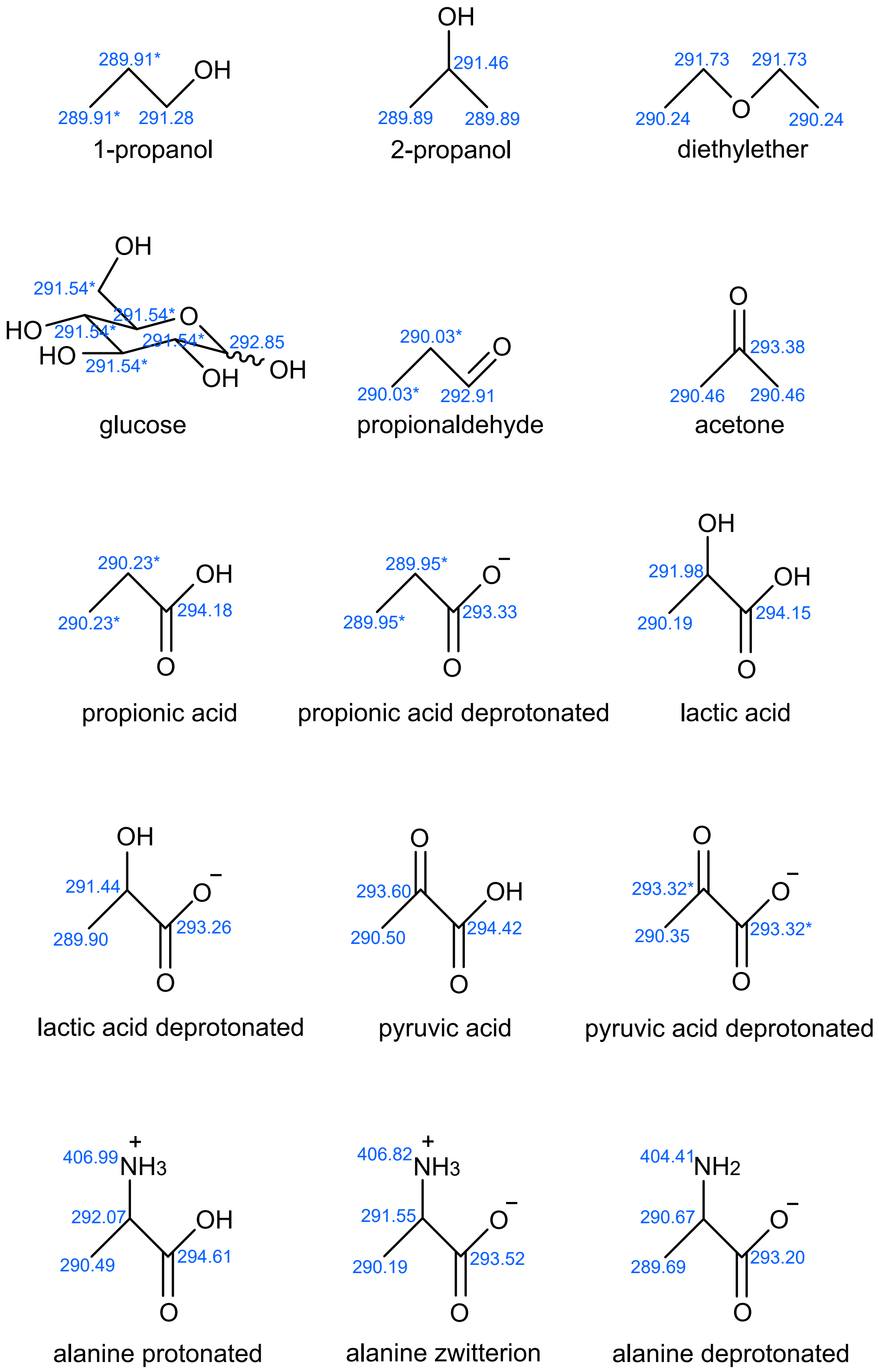

Figure S2: Overview of C 1s and N 1s binding energies (eV) measured from 0.5 M aqueous solutions of the respective compounds. Values denoted by an asterisk come from a single peak in the compound's spectrum.
