# Supplementary figures and images for: Electron spectroscopy for chemical analysis of liquids
Source: Chem Sci. 2026 Feb 4;17(12):6156–64. doi: 10.1039/d5sc09061j (PMC12869189; doi:10.1039/d5sc09061j)

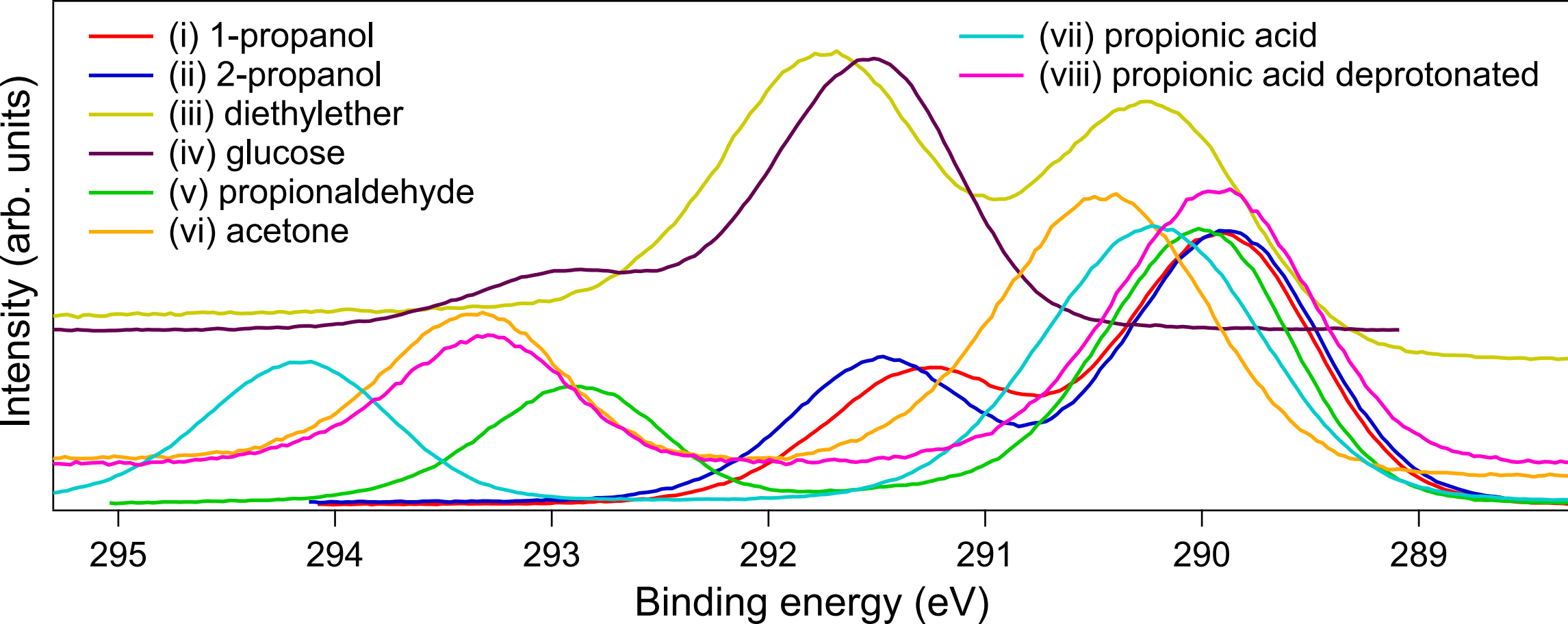

Supplement: SC-017-D5SC09061J-s002 [file SC-017-D5SC09061J-s002.zip › FigS1.pdf]

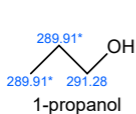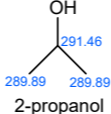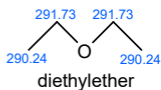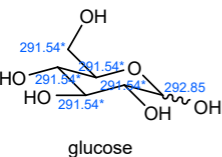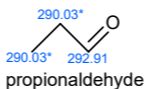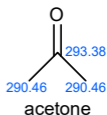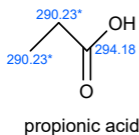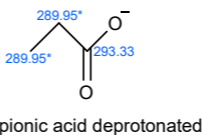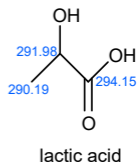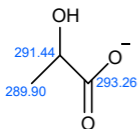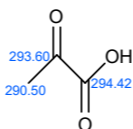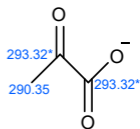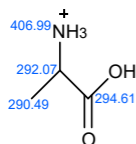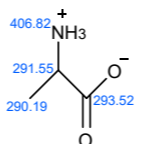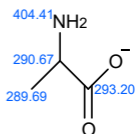

Supplement: SC-017-D5SC09061J-s002 [file SC-017-D5SC09061J-s002.zip › FigS2.pdf]
